# A Consensus Meeting on Expert Recommendations on Operating Specifications for Laparoscopic Radical Resection of Hilar Cholangiocarcinoma

**DOI:** 10.3389/fsurg.2021.731448

**Published:** 2021-11-23

**Authors:** Yongfu Xiong, Li Jingdong, Tang Zhaohui, Joseph Lau

**Affiliations:** ^1^Department of Hepatobiliary Surgery, Affiliated Hospital of North Sichuan Medical College, Nanchong, China; ^2^Institute of Hepato-Biliary-Pancreatic-Intestinal Disease, North Sichuan Medical College, Nanchong, China; ^3^Research Office of Hepato-Biliary-Pancreatic-Intestinal Disease, Affiliated Hospital of North Sichuan Medical College, Nanchong, China; ^4^Department of General Surgery, Wusheng County People's Hospital, Guang'an, China; ^5^Department of General Surgery, Xinhua Hospital, School of Medicine, Shanghai Jiao Tong University, Shanghai, China; ^6^Faculty of Medicine the Chinese University of Hong Kong, Prince of Wales Hospital, Shatin, Hong Kong SAR, China

**Keywords:** expert recommendations, operating specifications, laparoscopic radical resection, hilar cholangiocarcinoma, consensus meeting

## Abstract

**Background:** With advances in techniques and technologies, laparoscopic radical resection of hilar cholangiocarcinoma (HCCA) has gradually been carried out in major medical centers in China. Its feasibility and safety have been accepted by a group of Chinese surgical experts.

**Methods:** To standardize perioperative management of HCCA by using laparoscopic resectional approach, to ensure safety of the patient with standardized management, improve prognosis of the patient, and enable proper application and refinement of this surgical approach, the expert group on specifications for laparoscopic radical resection of HCCA in China organized a consensus meeting.

**Results:** Laparoscopic radical resection of HCCA is difficult and associated with high risks. Appropriate patients should be carefully selected and this surgical approach should be promoted gradually. The experts met and arrived at 16 recommendations on perioperative management of HCCA by using laparoscopic surgery. There were three recommendations on preoperative diagnosis and evaluation; one recommendation on surgical principles of treatment; one recommendation on indications and contraindications; one recommendation on credentialing, staffing, and equipment; nine recommendations on laparoscopic techniques in different stages of operation; and one recommendation on indications for conversion to open surgery.

**Conclusion:** Laparoscopic surgery for HCCA is still in the early phase of development. This consensus provides a clinical reference with the aim to promote and to facilitate its further development.

## Introduction

Hilar cholangiocarcinoma (HCCA) is a common malignant biliary tract tumor. Improvements in medical imaging have led to better diagnosis and staging of this disease. Radical resection is still the only treatment that can offer a chance of cure. However, the special anatomical location of HCCA, with its proximity to hepatic artery, portal vein, and caudate lobe makes excisional surgery extremely difficult (1, 2). Recent advances in minimally invasive surgery have attracted pioneer surgeons to perform laparoscopic radical resection of HCCA in selected patients. With gradual establishment of this operation on its feasibility, safety, and short-term treatment outcomes, this approach has now been gradually adopted by expert biliary surgeons in China as an alternative approach to open surgery on highly selected patients. To standardize the perioperative management of HCCA and the technical steps in laparoscopic radical resection of HCC, to ensure safety of the patient and improve prognosis, the expert group on specifications for laparoscopic radical resection of HCCA organized a consensus meeting for all the biliary expert surgeons in this field in China to formulate expert recommendations for laparoscopic radical resection of HCCA and its perioperative management.

An expert consensus meeting was held on April 19, 2021, during the Second Congress of the Hilar Cholangiocarcinoma Study Group of Surgeons. Experts in HCCA surgery were invited to participate in the meeting and to present specific issues with respect to laparoscopic surgery for HCCA including oncologic concerns, selection criteria, surgical techniques, and future aspects of this procedure. Presentations were followed by panel discussions and open discussions with the audience. After meeting, a first draft including summaries of the presentations and discussions was circulated to the panels, discussed, and edited. This document, including expert consensus statements, was formulated by all the attending experts in this field.

The categories of evidence used in this current expert recommendation are shown in Table 1 and the recommendation grades are given in Table 2.

**Table 1:** Level of evidence.

**Level (quality) of evidence**	**Requirements**
Class 1	•High-quality evidence from more than 1 RCT •Meta-analyses of high-quality RCTs •One or more RCTs corroborated by high-quality registry studies
Class 2	•Moderate-quality evidence from 1 or more RCTs •Meta-analyses of moderate-quality RCTs
Class 3	•Moderate-quality evidence from 1 or more well-designed, well-executed non-randomized studies, observational studies, or registry studies •Meta-analyses of such studies
Class 4	•Randomized or non-randomized observational or registry studies with limitations of design or execution •Meta-analyses of such studies •Physiological or mechanistic studies in human subjects
Class 5	•Consensus of expert opinion based on clinical experience

*RCTs, randomized clinical trials*.

**Table 2:** Recommendation grades for this current expert recommendation.

**Recommendation grade**	**Criteria**
Grade I	Strong recommendation
Grade II	Moderate recommendation
Grade III	Weak recommendation
Grade IV	No recommendation

## Preoperative Diagnosis, Preparation, and Evaluation of Laparoscopic Radical Resection of HCCA

### Key Points in Preoperative Diagnosis and Preparation

A comprehensive, effective, and complete evaluation should be carried out based on clinical symptoms/signs, laboratory findings, and medical imaging results (Table 3). The key findings used in preoperative diagnosis of open radical resection of HCCA should be the same as in laparoscopic surgery (1, 2).

**Table 3:** List of recommending preoperative examinations for hilar cholangiocarcinoma.

**List of examinations**	**Features**	**Category of recommendation**
B-ultrasound	Evaluates degree of tumor invasion. Doppler ultrasound is helpful to evaluate portal vein invasion.	II
CT (MDCT)	Thin-slice scan is helpful to show vascular invasion. It has advantages in defining tumor location, size, biliary obstruction level, liver atrophy, and three-dimensional imaging of blood vessels. It has a high accuracy in determining resectability.	I
MRI+MRCP	Provides high resolution of soft tissues, with adequate display of biliary system, and secondary changes to bile ducts. It has special values in evaluating the longitudinal extent of bile duct tumor.	I
ERCP/ENBD	Invasive investigations which can accurately show the whole bile duct. It can be used for preoperative drainage to reduce jaundice.	III
PTCD	First choice to reduce obstructive jaundice before surgery. It is not recommended as a diagnostic procedure.	II
Endoscopic ultrasound	Has certain value for tumors with associated bile duct stones or cystic dilatation of the bile duct.	III
PET-CT	Is not recommended for early or intermediate stages of tumors, but has value to determine distant metastases.	II
Laparoscopic exploration	Useful for clinical staging of tumors.	III

#### Expert Recommendation 1

Multidetector CT (MDCT) (class 1, grade I) and magnetic resonance cholangiopancreatography (MRCP) (class 1, grade I) are recommended as the most important investigations because they can clearly delineate tumor location, size, level of biliary obstruction, blood vessel invasion, and atrophy of different parts of liver. Before surgery, a proper drainage procedure, such as percutaneous transhepatic cholangiodrainage (PTCD) or endoscopic nasobiliary drainage (ENBD), should be performed to relieve jaundice and any obstruction (class 1, grade I). Portal vein embolization (PVE) can be used to increase the size of future liver remnant (FLR) and has been shown to be effective in inducing liver hypertrophy with minimal risks. Biliary drainage should be established before PVE in patients with biliary dilatation in FLR (class 2, grade I).

### Importance of Classifications and Stagings for Laparoscopic Radical Resection of HCCA

**Figure 1:**
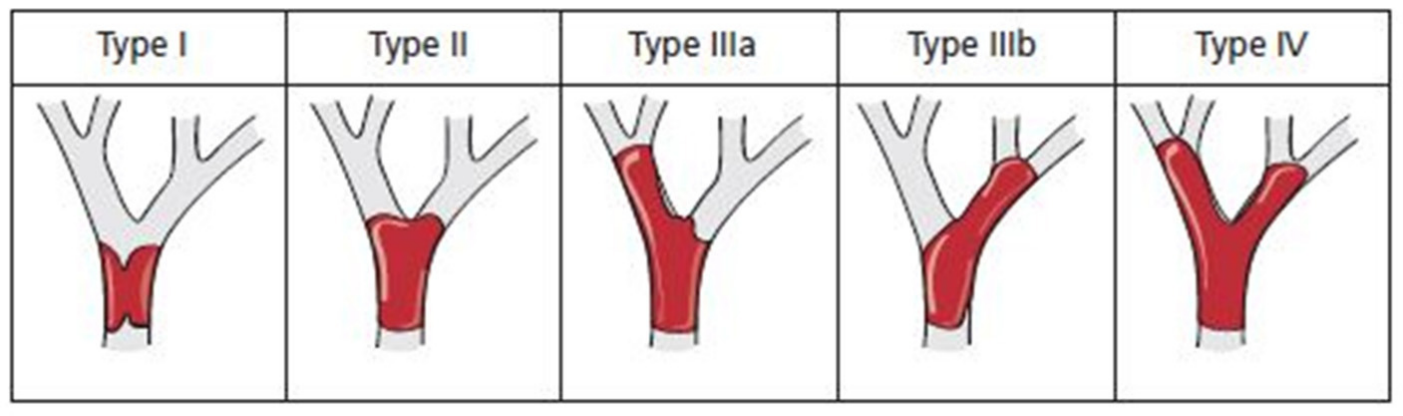
Schematic diagram of the Bismuth–Corlette classification of hilar cholangiocarcinoma (HCCA).

Classifications and stagings were considered to be of extreme importance in determining resectability of the tumor (Table 4). The Bismuth–Corlette classification is recommended for HCCA (class 1, grade I). Based on the level and extent of invasion of the biliary tract, this classification was considered by the experts in providing a sound basis to determine local extent of resection and degree of combined liver resection before surgery (Figure 1).

**Table 4:** Preoperative classification and staging for hilar cholangiocarcinoma.

**Classification or staging**	**Application characteristics**	**Category of recommendation**
Bismuth-Corlette classification	The most widely used clinical classification. It considers the level and scope of biliary invasion but does not consider vascular invasion or lymphatic or distant metastasis.	I
AJCC/UICC	Can be used to assess local or distant metastasis with guiding significance on prognosis of surgical treatment of tumors.	I
MSKCC staging	Has value as it includes vascular invasion and liver atrophy and is recommended to assist clinical decision	II
The international cholangiocarcinoma working group staging	Combines multiple types of classification and staging for comprehensive assessment but lacks good clinical trials with large samples to support.	II

The American Joint Committee on Cancer (AJCC) and the Union for International Cancer Control (UICC) tumor–node–metastasis (TNM) stagings are based on comprehensive analysis of postoperative pathological findings. They should be used in predicting prognosis and postoperative survival of patients. The Memorial Sloan Kettering Cancer Center (MSKCC) staging (class 2, grade II) should be used to systematically evaluate blood vessel invasion, lymph node metastasis, liver atrophy, and distant metastasis.

#### Expert Recommendation 2

The Bismuth–Corlette classification (class 1, grade I) should be used to make a preliminary decision on the surgical method used. It is recommended to supplement the Bismuth–Corlette classification with the AJCC- and the UICC-related TNM stagings (class 1, grade I) and the International Cholangiocarcinoma Working Group Staging (class 2, grade II) to predict resectability of HCCA and long survival outcomes after treatment (3).

### Preoperative Evaluation

Preoperative evaluation for laparoscopic radical resection of HCCA should be the same as in open surgery and should cover the following:

(1) Evaluation of degree of bile duct involvement: This is the primary target in preoperative evaluation. Magnetic resonance cholangiopancreatography combined with MDCT should be used to evaluate the degree of bile duct invasion, with visual display of the structural characteristics of the whole biliary system, length and extent of tumor involvement, and depth of invasion of bile duct wall (1, 2).
(2) Evaluation of adjacent vascular invasion: Preoperative imaging examinations combined with three-dimensional reconstruction should be used to determine whether adjacent blood vessels are invaded and the location and extent of invasion are important in determining resectability of the tumor.
(3) Three-dimensional CT reconstruction, visualization, and assessment: First, this technique can be used to display the anatomies of intrahepatic bile ducts and blood vessels from multiple angles and multiple levels, thus helping to assess any anatomical anomalies to avoid unnecessary injuries during operation and to better protect the structures and function of the remnant liver (4). Second, this assessment can help to quantitatively analyze tumor volume and volumes of each liver segment and its combination. It can also be used to carry out simulation surgery in planning operations and in selecting an optimal liver transection plane and to calculate residual liver volumes (3, 4).
(4) Evaluation of lymph node metastasis: PET-CT should be used to evaluate lymph node metastasis and extent of involvement.

#### Expert Recommendation 3

Preoperative MDCT (class 1, grade I) and MRCP (class 1, grade I) should be used routinely to assess the extent of bile duct and blood vessel invasion. Three-dimensional CT reconstruction, visualization, and assessment system (class 2, grade I) should be used routinely to evaluate anomalies of bile ducts and blood vessels and to calculate residual liver volumes. PET-CT is recommended to detect possible lymph node metastasis (class 2, grade II).

## Indications and Treatment Principles for Laparoscopic Radical Resection of HCCA

### Indications and Contraindications

The indications for laparoscopic radical resection of HCCA should be more stringent than in open surgery. Preoperative CT, MRCP, CT angiography, or magnetic resonance angiography should be used to clarify the relationship between the tumor and the hepatic artery and portal vein and to determine whether there is any invasion. The indications include the Bismuth–Corlette type I, type II, and some types III and IV tumors with no portal vein or hepatic artery invasion. In addition to all the contraindications to open radical resection of HCCA, the contraindications should also include intolerance to prolonged pneumoperitoneum, failure to establish pneumoperitoneum, extensive abdominal adhesions, difficulties in dissecting or exposing the lesion, extensive tumor invasion of portal vein or common hepatic artery, difficulties in obtaining adequate laparoscopic view, or presence of portal hypertension resulting in high surgical risks (4).

#### Expert Recommendation 4

The indications and contraindications should be strictly followed to select suitable patients for laparoscopic resection of HCCA. The Bismuth–Corlette type I and type II can be successfully resected and reconstructed by using laparoscopic surgery in expert hands. Laparoscopic surgery is also feasible for some Bismuth–Corlette types III and IV. For any Bismuth–Corlette type with tumor invasion of portal vein, common hepatic artery and their branches and vascular resection and reconstruction are recommended, if technically feasible. Otherwise, conversion to open surgery is recommended (class 1, grade I).

### Principles of Treatment

The principles of laparoscopic radical resection of HCCA should be the same as in open radical resection of HCCA. The current standard of open surgery includes partial hepatectomy with en bloc resection of the tumor-invaded bile duct, regional lymph node and nerve dissection, and hepatic duct–jejunum Roux-en-Y anastomosis, with emphasis on complete tumor resection (R0 resection) with negative margins, including the invaded bile duct with the adjacent tissues and restoration of biliary-intestinal continuity of the functional residual liver remnant (4, 5). Anatomical liver resection is the standard procedure for HCCA. To achieve R0 resection, it is recommended to carry out intraoperative frozen sections to confirm negative proximal and distal bile duct resection margins (5–8).

#### Expert Recommendation 5

The principles of laparoscopic radical resection of HCCA should be the same as in open surgery. However, due to the limitations of laparoscopic operations in intraoperative assessment of liver resection margins, it is recommended to routinely perform anatomical major liver resections, e.g., hemihepatectomies or trisectionectomies combined with caudate lobe resection. Limited liver resections aiming to preserve functional liver parenchyma with local excision of adjacent liver parenchyma are not recommended (class 1, grade I).

## Requirements for Hospital Credentialing on Adequate Staffing and Surgical Equipment for Laparoscopic Radical Resection of HCCA

In the early phase of promoting this surgery, laparoscopic radial resection of HCCA should only be confined to credentialed hospitals with good experience in major laparoscopic surgeries and with adequate staffing to form a fixed surgical team consisting of a chief surgeon, a first assistant, a scope operator, a scrub nurse, and an anesthesiologist. This surgical team should be proficient in carrying out complex laparoscopic operations, including laparoscopic liver resection and laparoscopic pancreaticoduodenectomy, and has crossed the required learning curves for these operations (recommended number of cases >50 for each of these types of surgery) (9). For surgical equipment, in addition to the conventional laparoscopic equipment and instruments, a good quality laparoscopic system, a LigaSure, ultrasonic knife, or Cavitron Ultrasonic Surgical Aspirator (CUSA) is recommended.

During the process of development and promotion of this surgery, patients should be highly selected during the learning curve to gradually step up from less complex to more complex operations. The surgical team must have adequate experience in open radical resection of HCCA and the ability to complete the operation when conversion to open surgery is required and to deal with any complications, which may arise out of the laparoscopic operations.

### Expert Recommendation 6

Laparoscopic radical resection of HCCA should only be carried out in large medical centers with adequate experience in laparoscopic hepatectomy and laparoscopic pancreaticoduodenectomy, after passing the learning curves, and with adequate experience in complex laparoscopic hepatectomy and bile duct reconstruction (class 1, grade I). There should be a fixed team in carrying out laparoscopic resection of HCCA (class 1, grade I). In the early phase of development, patients with less complex pathologies should be selected. There should be a gradual move to operate on more complex pathologies after accumulation of adequate operative experience (class 1, grade I).

## Surgical Procedures

### Establishment of Operating Ports in Laparoscopic Radical Resection of HCCA

**Figure 2:**
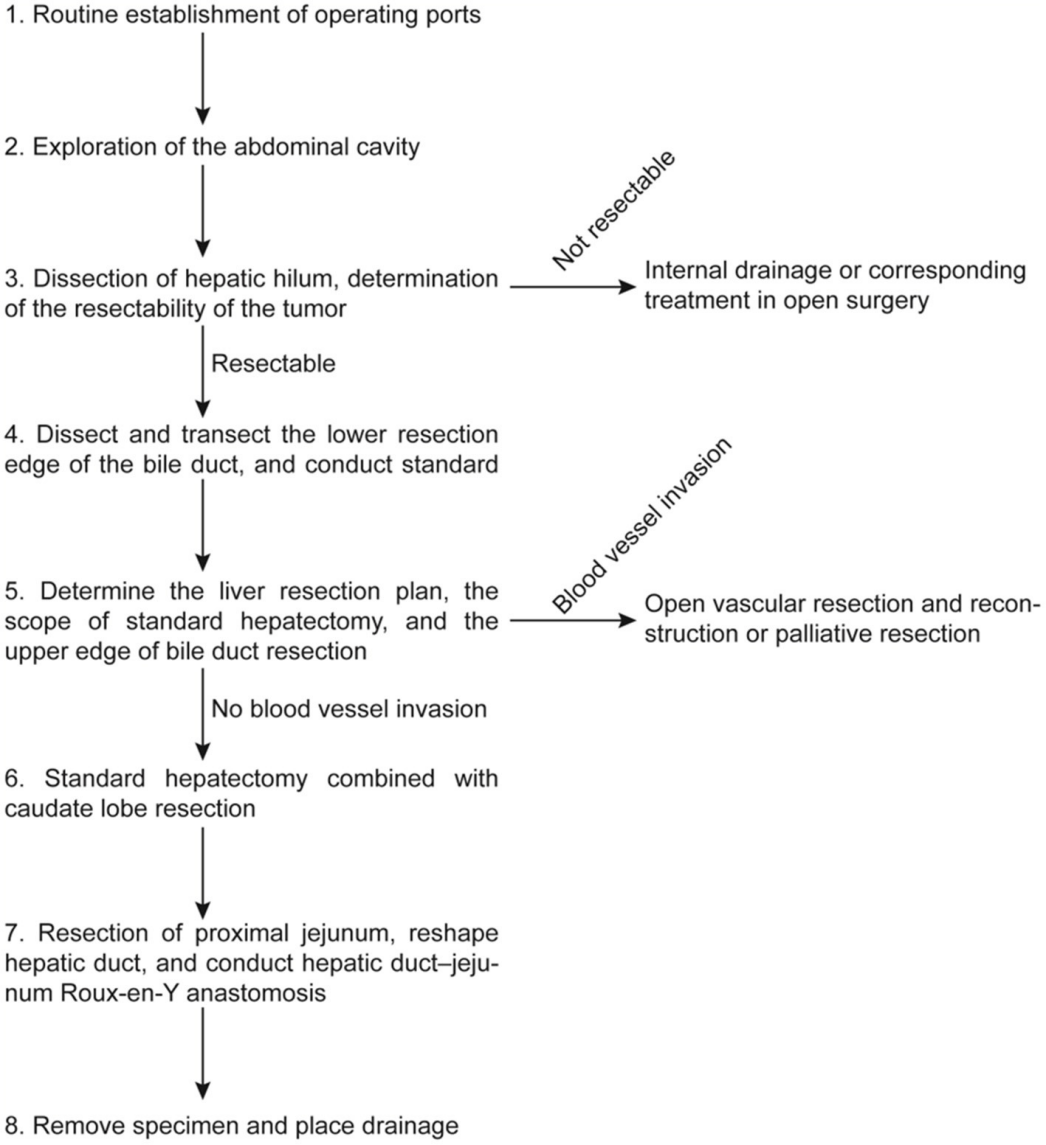
Operation flowchart. This operation procedure was based on the recommendations of the experts who participated in the consensus meeting to provide a clinical reference for less experienced surgeons. Surgeons should carry out his/her operation procedures according to the specific situations during operation.

Generally, the five-port technique and split-leg position of patient are used. The port sites are recommended to center on the hepatic hilum in a V-shaped distribution. The specific port sites should be determined according to the planned operative procedure, taking into consideration of the requirements of liver resection and biliary reconstruction. Ancillary ports should be added when needed (Figure 2).

#### Expert Recommendation 7

When establishing the operating ports, the camera port is recommended to be placed under the umbilicus to facilitate reconstruction and anastomosis. The remaining ports should be centered on the hepatic hilum and should be distributed in a V shape. However, specific port layout should be individualized according to the planned operation. Operating ports should be added if necessary to facilitate surgery and to speed up the operative process (class 1, grade I).

### Procedures for Intraoperative Laparoscopic Exploration

Routine exploration should be conducted to access whether there is any intraperitoneal metastasis. The hilar region should be dissected to determine the size, location, and extent of the hilar tumor. The depth of tumor involvement of bile duct, the relationship between the tumor with the portal vein and hepatic artery, the extent of vascular invasion, if any, and the involvement of caudate lobe should be assessed to reach to a preliminary decision on the possibility of radical resection and the extent of resection (1, 10–13) (Figure 2).

#### Expert Recommendation 8

Routine laparoscopic exploration should be carried out to exclude peritoneal metastases and small liver metastases. Laparoscopic ultrasonography should be used routinely to increase the accuracy of assessment (class 1, grade I).

### Extent of Regional Lymphatic and Nerve Plexus Dissection in Laparoscopic Radical Resection of HCCA

The extent of routine lymphatic and nerve plexus dissection should include the hilar region, hepatoduodenal ligament, tissues around the common hepatic artery, and lymph nodes and nerve plexus behind the head of pancreas. Different surgical approaches can be used according to the usual practice of the surgeon, the extent and location of the tumor, and the approach used in the laparoscopic operation. A combined left- and right-sided approach is recommended.

Left-sided approach: The omental bursa (lesser sac) is opened, followed by dissection of lymph nodes around the common hepatic artery (8a, 8p) and lymph nodes around the celiac trunk. The common hepatic artery and the gastroduodenal artery (the latter can be transected if needed to better expose the portal vein) are slinged and the right gastric artery is transected. Skeletonized dissection of all the lymph nodes, fat, and nerve tissues in the hepatoduodenal ligament (12h, 12e, 12b, 12a, and 12p) then follows. The 13a lymph nodes in the posterior edge of the pancreas are then dissected, together with the lymph nodes adjacent to the abdominal aorta (no. 16).

Right-sided approach: The peritoneum on the side of duodenal peritoneum is opened to dissect the 13a lymph nodes on the posterior edge of pancreas, which should be immediately sent for intraoperative frozen section. If positive, no. 16 lymph nodes need to be removed and if no. 16 lymph nodes are positive, radical resection should be abandoned. If negative, all the tissues in the hepatoduodenal ligament, with the exception of the hepatic artery and portal vein are removed en bloc. The lymph nodes around the common hepatic artery (8a, 8p) and those around the celiac trunk, together with the surrounding fatty tissues, are then dissected (14–16) (Figure 2).

#### Expert Recommendation 9

Lymph node dissection should be standardized. Attention should be paid in protecting the surrounding blood vessel walls. There is no special rule for the sequence of dissection. Appropriate sequences of regional lymph node and nerve plexus dissection should be based on the intraoperative findings, the laparoscopic approaches used, and the usual practice of the operating surgeon (class 1, grade I).

### Important Technical Points to Achieve Laparoscopic R0 Resection of HCCA

During laparoscopic surgery, the relatively small operating space of the hilum can be zoomed in to allow anatomical structures of the hilum to be displayed more clearly and three-dimensionally.

To dissect the hepatic hilum, the common bile duct is first isolated, ligated at the upper edge of the pancreas, and then transected. The lower resection margin of the bile duct should routinely be sent for frozen section examination. Bile duct dissection is then continued from a caudal to cranial direction. The skeletonized hepatic artery and portal vein are suspended to facilitate subsequent procedures. After assessing the extent of tumor invasion of the bile duct for resectability, the hepatic artery, portal vein, and their branches are dissected to determine whether the tumor has any vascular invasion. If the blood vessel supplying the hemiliver, which is planned to be preserved is found to be invaded, the surgery should be converted to open surgery. For any difficulties in determining the upper extent of biliary involvement by tumor, the liver parenchyma can be split to reveal the upper extent of the HCCA. When the upper extent of tumor has far exceeded the U point or P point, palliative surgery in reducing jaundice can be carried out. For resectable HCCA, the hepatic duct on the side of the liver to be preserved should be resected and the upper resection margin of the bile duct should be sent for frozen section examination. Repeated frozen sections and pathological examinations during the operation should be done to ensure negative resection margins with R0 resection (10, 15) (Figure 2).

#### Expert Recommendation 10

The hilar structures should be dissected to clarify whether the tumor has invaded blood vessels and to determine the extent of anatomical liver resection and the extent of bile duct resection based on preoperative and intraoperative findings. During the operation, frozen sections should be repeatedly conducted to assure negative margins of bile ducts, blood vessels, and liver to achieve R0 resection (class 1, grade I).

### Important Technical Points in Handling the Caudate Lobe

In laparoscopic radical resection of HCCA, when combined with anatomical hemihepatectomy, extended hemihepatectomy, or trisectionectomy, the short hepatic veins can be clearly seen laparoscopically, thus facilitating en bloc resection of the caudate lobe.

Turning the liver and Spigelian lobe to the right allows good exposure for ligation and division of the short hepatic veins on the left side of the inferior vena cava upto the suprahepatic portion. The short hepatic veins on the right of the inferior vena cava are dealt with using the right-sided approach with dissection from bottom to top. As for the short hepatic veins near the confluence of hepatic veins, it is safer to treat them after splitting the liver parenchyma down to the inferior vena cava (15–17). The thin backflow branches of the short hepatic veins can be transected with an ultrasonic knife or LigaSure and the larger branches can be transected after ligation. If necessary, they can be transected and sutured. After the portal vein branches to the caudate lobe are exposed and disconnected, the whole caudate lobe together with the resected portion of liver containing the resected bile ducts with the tumor can be resected en bloc (18, 19) (Figure 2).

#### Expert Recommendation 11

A combined approach to mobilize the caudate lobe from left to right and from right to left to deal with the short hepatic veins should be used. After complete mobilizing the caudate lobe, the portion of the liver to be resected together with the bile ducts containing the tumor can be resected en bloc (class 2, grade II).

### Important Technical Points on Liver Resection in Laparoscopic Radical Resection of HCCA

The important technical points on liver resection in laparoscopic radical resection of HCCA are roughly the same as those in laparoscopic or open hepatectomy for other liver tumors or hepatolithiasis. The main difference lies in that in laparoscopic hepatectomy for HCCA, special efforts should be made to combine preoperative imaging (including three-dimensional reconstruction) and intraoperative findings to determine the plane and extent of liver resection (e.g., left or right hemihepatectomy, extended left or right hemihepatectomy) plus caudate lobe resection.

When right hepatectomy plus total caudate lobectomy is required for a patient with HCCA, the surgeon should choose instruments for liver parenchymal transection that he/she is most familiar with (such as an ultrasound knife or a laparoscopic CUSA). After ligating and dividing the right hepatic artery and the right portal vein, the liver parenchyma can be transected from a caudal to cranial direction along the ischemic line on the liver surface. Small blood vessels (<3 mm) on the transected liver raw area can be cauterized, while larger and thicker branches are transected after ligation. The right hepatic vein can be treated with an endoscopic cutting and vascular closure device (10, 20–22). The short hepatic veins are then transected along the inferior vena cava. After dividing the hepatocaval ligament and mobilizing the caudate lobe, the whole resected specimen can be removed en bloc (23, 24).

Left hepatectomy plus total caudate lobectomy is technically similar to the right-sided operation, although the operation is relatively easier. Patients with the Bismuth–Corlette type IV HCCA should be strictly and carefully selected. The surgical operation should be chosen based on the location of tumor, the extent of bile duct invasion, and any atrophy affecting the liver. Extended right hepatectomy, extended left hepatectomy, or resection of right or left hemiliver can be carried out in well-selected patients. A right-sided liver resection is safer than a left-sided liver resection because the right hepatic artery runs behind the common hepatic duct and is more susceptible to invasion by tumor and there is a longer length of the left hepatic duct than the right hepatic duct (25, 26) (Figure 2).

#### Expert Recommendation 12

Accurate preoperative and intraoperative decision on the extent of liver resection and the plane of liver transection are the key technical points for laparoscopic radical resection of HCCA. In the process of liver resection, tumor-free resection margins of bile ducts, blood vessels, and liver planes should be achieved. In the presence of severe liver cirrhosis or insufficient preoperative jaundice reduction, combined major hepatectomy aiming to achieve R0 resection should not be aggressively carried out. Palliative surgery should be used as an alternative treatment to ensure safety of the patient (class 2, grade II).

### Important Technical Points on Hepatic Duct–Jejunum Anastomosis for Laparoscopic Radical Resection of HCCA

The technique of laparoscopic hepatic duct–jejunum anastomosis is more difficult and more demanding when compared with open surgery. When the open end of the hepatic duct is relatively large and its position is shallow, anastomosis is easier; otherwise, it can be technically very difficult. The number of hepatic duct openings on the liver remnant would depend on the plane and extent of resection.

For the hepatic duct–jejunum anastomosis, whether the anastomosis should be completed antecolic or retrocolic would depend on the body build of the patient and the findings of the operation. Tension should be avoided. Before the anastomosis, the hepatic duct should be properly shaped and anastomosed to a Roux-en Y jejunal loop. Continuous suturing is recommended for the posterior wall and for the anterior wall either continuous or intermittent suturing can be used, according to the size, position, and angle of the bile duct (10, 21, 27). For type IIIb HCCA, resection is relatively easier, but the right hepatic duct to jejunum anastomosis is more difficult. Right hemihepatectomy plus caudate lobectomy for type IIIa HCCA is technically more difficult, but the hepatic duct and jejunum reconstruction are relatively easier (28–31). The intestinal–intestinal anastomosis should be more than 45–60 cm away from the biliary–enteric anastomosis. If necessary, an external drainage decompression tube can be placed across the hepatic–enteric anastomosis (Figure 2).

#### Expert Recommendation 13

For HCCA, bile duct resection should be combined with major liver resection plus caudate lobe resection. When the number of bile ducts left in the liver remnant is large, temporary stay stitches can help to expose the bile duct openings to improve the quality of the cholangiojejunostomy. For those patients with difficult anastomoses, conversion to open surgery by using a median small incision to construct a difficult anastomosis helps. Placement of an external drainage decompression tube can help in better healing of the anastomosis. For patients who cannot undergo major liver resection, palliative resection or bile duct–jejunum bypass should be considered (class 1, grade I).

### Important Technical Points in Managing the Raw Transected Liver Surfaces, Placement of Drainage Tubes, and Removal of Specimens

After liver transection, the raw liver surface should be carefully inspected. Small biliary leakage and bleeding points should be closed by using suitable sutures (10, 22, 30, 31). The resected specimen should be placed into a specimen bag and removed through a small suprapubic transverse incision in the lower abdomen. A drainage tube should be routinely placed below the biliary–enteric anastomosis and on the raw liver area. The specimen should be routinely examined histopathologically to determine the tumor location and the extent of tumor involvement (Figure 2).

#### Expert Recommendation 14

The transected raw liver surface should be careful dealt with to stop all the bleeding and bile leakage points. Drainage tubes should be placed posterior to the biliary–enteric anastomosis and on the raw liver area. Care should be taken to ensure that the drainage tubes are not obstructed by kinking (class 1, grade I).

### Laparoscopic Radical Resection for Patients With HCCA Requiring Vascular Resection and Reconstruction

Hilar cholangiocarcinoma resection, when combined with vascular resection and reconstruction, can lead to a higher chance of achieving R0 resection for patients with vascular invasion, thereby improving survival of these patients (32, 33). Invasion of blood vessels significantly increases the difficulty of laparoscopic radical resection of HCCA. Resection and reconstruction of hilar blood vessels under laparoscopic surgery are technically difficult. Once invasion of portal vein or hepatic artery supplying the planned-preserved hemiliver or the main trunks is found during operation, conversion to open surgery is recommended (Figure 2).

#### Expert Recommendation 15

If vascular resection and reconstruction are necessary due to invasion found on the hemiliver, which is planned to be preserved, prompt conversion to laparotomy is recommended (class 1, grade I).

## Indications for Conversion to Laparotomy

Conversion to laparotomy should be carried out for the following conditions: uncontrollable bleeding; intolerance to pneumoperitoneum; difficulties in exposing or resecting the lesion; intraoperative detection of invasion of main vascular trunk or blood vessels of the side of the liver to be preserved; multiple open ends of the transected bile duct; difficulty in bile duct reshaping and biliary–intestinal anastomosis; unsatisfactory anastomosis; or difficulty/failure to continue the operation under laparoscopy. Timely conversion to laparotomy can reduce serious complications and is beneficial to patients.

### Expert Recommendation 16

Timely conversion to laparotomy to ensure safety of the patient should be the primary consideration for laparoscopic surgeons (class 1, grade I).

## Summary

Laparoscopic radical resection of HCCA is technically difficult and it is still associated with high operative risks. Appropriate patients should be carefully selected. This surgical approach should be promoted carefully and gradually. It is recommended that only hepatobiliary and pancreatic laparoscopic surgery centers in large general hospitals with adequate experience in laparoscopic operations in other less complex hepaticopancreatobiliary operations should be acquired before attempting to develop this operation to come up with a safe and feasible operation for others to follow. It is also recommended to conduct clinical studies on this operation, focusing on safety of the patient and treatment effectiveness after standardizing the surgical procedures involved in this operation. The ultimate aim is to improve the quality and quantity of life for patients with HCCA.

## Data Availability Statement

The original contributions presented in the study are included in the article/supplementary material, further inquiries can be directed to the corresponding author/s.

## Author Contributions

All authors listed have made a substantial, direct, and intellectual contribution to the work and approved it for publication.

## Funding

Doctoral research start-up fund of North Sichuan Medical College (201901 to YX) Project of multicentre clinical research of Shanghai Jiaotong University School of Medicine (DLY201807, to TZ). We declare that all sources of funding received for the research being submitted.

## Conflict of Interest

The authors declare that the research was conducted in the absence of any commercial or financial relationships that could be construed as a potential conflict of interest.

## Publisher's Note

All claims expressed in this article are solely those of the authors and do not necessarily represent those of their affiliated organizations, or those of the publisher, the editors and the reviewers. Any product that may be evaluated in this article, or claim that may be made by its manufacturer, is not guaranteed or endorsed by the publisher.

## References

[1] China A-cAO. Guideline for the diagnosis and therapy of hilar cholangiocarcinoma (2015). Chin J Hepatobil Surg. (2015). 21:505–11. 10.3760/cma.j.issn.1007-8118.2015.08.00126172136

[2] Sharpe SM Talamonti MS Wang CE Prinz RA Roggin KK Bentrem DJ . Early national experience with laparoscopic pancreaticoduodenectomy for ductal adenocarcinoma: a comparison of laparoscopic pancreaticoduodenectomy and open pancreaticoduodenectomy from the national cancer data base. J Am Coll Surg. (2015) 221:175–84. 10.1016/j.jamcollsurg.2015.04.021 26095569

[3] Deoliveira ML Schulick RD Nimura Y Rosen C Gores G Neuhaus P . New staging system and a registry for perihilar cholangiocarcinoma. Hepatology. (2011) 53:1363–71. 10.1002/hep.24227 21480336

[4] Zhang J Qiao QL Guo XC Zhao JX. Application of three-dimensional visualization technique in preoperative planning of progressive hilar cholangiocarcinoma. Am J Transl Res. (2018) 10:1730–5. 30018714 PMC6038071

[5] Cho A Yamamoto H Kainuma O Muto Y Yanagibashi H Tonooka T . Laparoscopy in the management of hilar cholangiocarcinoma. World J Gastroenterol. (2014) 20:15153–7. 10.3748/wjg.v20.i41.15153 25386064 PMC4223249

[6] Zhang CW Liu J Hong DF Wang ZF Hu ZM Huang DS . Pure laparoscopic radical resection for type IIIa hilar cholangiocarcinoma. Surg Endosc. (2018) 32:1581–2. 10.1007/s00464-017-5741-4 28779241

[7] Zhi-qiang H. Management of hilar cholangiocarcinoma: review of a 25-year experience. Chin J Digest Surg. (2010). 9:161–4. 10.3760/cma.j.issn.1673-9752.2010.03.001

[8] Öter V Özer I Dalgiç T Binarbaşi C Ulaş M Bostanci EB. Results of positive proximal margin after resection for hilar cholangiocarcinoma: an analysis of 42 cases. Turk J Gastroenterol. (2019) 30:88–94. 10.5152/tjg.2018.17752 30301710 PMC6389298

[9] Tan CL Zhang H Peng B Li KZ. Outcome and costs of laparoscopic pancreaticoduodenectomy during the initial learning curve vs laparotomy. World J Gastroenterol. (2015) 21:5311–9. 10.3748/wjg.v21.i17.5311 25954105 PMC4419072

[10] Yin Xinming LY Wei C Yifei W Yi L Siwei Z Chunhong L. Clinical application value of total laparoscopic radical resection of IV-type hilar choangiocarcinoma: video attached. Chin J Hepat Surg. (2018) 7:110−4. 10.3877/cma.j.issn.2095-3232.2018.02.007

[11] Puntambekar S Sharma V Kumar S Mitkare S Joshi G Parikh H. Laparoscopic management of hilar cholangiocarcinoma: a case report. Ind J Surg. (2016) 78:57–9. 10.1007/s12262-015-1345-1 27186042 PMC4848218

[12] Ito F Agni R Rettammel RJ Been MJ Cho CS Mahvi DM . Resection of hilar cholangiocarcinoma: concomitant liver resection decreases hepatic recurrence. Ann Surg. (2008) 248:273–9. 10.1097/SLA.0b013e31817f2bfd 18650638

[13] Association for Medical and Healthcare; Pancreas Minimally Invasive Group in Pancreatic Diseases Committee of Chinese Research Hospital Association; Pancreas Minimally Invasive Group in Pancreatic Cancer Committee of Chinese Anti-Cancer Association Expert consensus of laparoscopic pancreaticoduodenectomy(postscript of operation process and main steps). Zhonghua Wai Ke Za Zhi. (2017). 55:335–9. 10.3760/cma.j.issn.0529-5815.2017.05.004 28464571

[14] Mayo SC Austin DF Sheppard BC Mori M Shipley DK Billingsley KG. Evolving preoperative evaluation of patients with pancreatic cancer: does laparoscopy have a role in the current era? J Am Coll Surg. (2009) 208:87–95. 10.1016/j.jamcollsurg.2008.10.014 19228509

[15] Yi B Zhang BH Zhang YJ Jiang XQ Zhang BH Yu WL . Analysis of the relation between surgery and prognosis of hilar cholangiocarcinoma. Zhonghua Wai Ke Za Zhi. (2005) 43:842–5. 10.3760/j:issn:0529-5815.2005.13.004 16083598

[16] Molina V Sampson J Ferrer J Sanchez-Cabus S Calatayud D Pavel MC . Klatskin tumor: diagnosis, preoperative evaluation and surgical considerations. Cir Esp. (2015) 93:552–60. 10.1016/j.cireng.2015.07.002 26298684

[17] Han IW Jang JY Kang MJ Kwon W Park JW Chang YR . Role of resection for Bismuth type IV hilar cholangiocarcinoma and analysis of determining factors for curative resection. Ann Surg Treat Res. (2014) 87:87–93. 10.4174/astr.2014.87.2.87 25114888 PMC4127903

[18] Gumbs AA Jarufe N Gayet B. Minimally invasive approaches to extrapancreatic cholangiocarcinoma. Surg Endosc. (2013) 27:406–14. 10.1007/s00464-012-2489-8 22926892

[19] Ercolani G Zanello M Grazi GL Cescon M Ravaioli M Del Gaudio M . Changes in the surgical approach to hilar cholangiocarcinoma during an 18-year period in a Western single center. J Hepatobiliary Pancreat Sci. (2010) 17:329–37. 10.1007/s00534-009-0249-5 20464563

[20] Bryant R Laurent A Tayar C Cherqui D. Laparoscopic liver resection-understanding its role in current practice: the Henri Mondor Hospital experience. Ann Surg. (2009) 250:103–11. 10.1097/SLA.0b013e3181ad6660 19561476

[21] Hidalgo E Asthana S Nishio H Wyatt J Toogood GJ Prasad KR . Surgery for hilar cholangiocarcinoma: the Leeds experience. Eur J Surg Oncol. (2008) 34:787–94. 10.1016/j.ejso.2007.10.005 18036765

[22] Zheng S Zhenping HE Dong J Wang S Bie P Cai J . Twenty-year experience in surgical treatment of hilar cholangiocarcinoma. Chin J Gen Surg. (2001) 10:6–10. 10.3969/j.issn.1005-6947.2001.01.00311269010

[23] Shu-you PJ-tL. Choice of surgical procedures for hilar cholangiocarcinoma. J Clin Surg. (2006). 14:70−2. 10.3969/j.issn.1005-6483.2006.02.005

[24] Jang JY Kim SW Park DJ Ahn YJ Yoon YS Choi MG . Actual long-term outcome of extrahepatic bile duct cancer after surgical resection. Ann Surg. (2005) 241:77–84. 10.1097/01.sla.0000150166.94732.88 15621994 PMC1356849

[25] Palanivelu C Jani K Senthilnathan P Parthasarathi R Rajapandian S Madhankumar MV. Laparoscopic pancreaticoduodenectomy: technique and outcomes. J Am Coll Surg. (2007) 205:222–30. 10.1016/j.jamcollsurg.2007.04.004 17660068

[26] Kendrick ML Cusati D. Total laparoscopic pancreaticoduodenectomy: feasibility and outcome in an early experience. Arch Surg. (2010). 145:19–23. 10.1001/archsurg.2009.243 20083750

[27] Yu H Wu SD Chen DX Zhu G. Laparoscopic resection of Bismuth type I and II hilar cholangiocarcinoma: an audit of 14 cases from two institutions. Dig Surg. (2011) 28:44–9. 10.1159/000322398 21293131

[28] Lu Z Wang DD. Operation treatment method of Bismuth-Corlette III, IV hilar cholangiocarcinoma. Zhonghua Wai Ke Za Zhi. (2016) 54:488–91. 10.3760/cma.j.issn.0529-5815.2016.07.003 27373472

[29] Machado MAC Makdissi FF Surjan RC Mochizuki M. Laparoscopic resection of hilar cholangiocarcinoma. J Laparoendosc Adv Surg Tech A. (2012) 22:954–6. 10.1089/lap.2012.0339 23101791

[30] Han HS Cho JY Yoon YS Hwang DW Kim YK Shin HK . Total laparoscopic living donor right hepatectomy. Surg Endosc. (2015) 29:184. 10.1007/s00464-014-3649-9 24993170

[31] Rassam F Roos E van Lienden KP van Hooft JE Klümpen HJ van Tienhoven G . Modern work-up and extended resection in perihilar cholangiocarcinoma: the AMC experience. Langenbecks Arch Surg. (2018) 403:289–307. 10.1007/s00423-018-1649-2 29350267 PMC5986829

[32] Li JD Zhao ZL. Current status of laparoscopic techniques in the surgical treatment of biliary carcinoma. Zhonghua Wai Ke Za Zhi. (2018) 56:338–41. 10.3760/cma.j.issn.0529-5815.2018.05.004 29779308

[33] Lee Y Choi D Han S Han IW Heo JS Choi SH. Comparison analysis of left-side versus right-side resection in bismuth type III hilar cholangiocarcinoma. Ann Hepatobiliary Pancreat Surg. (2018) 22:350–8. 10.14701/ahbps.2018.22.4.350 30588526 PMC6295382

